# Weakly supervised localization model for plant disease based on Siamese networks

**DOI:** 10.3389/fpls.2024.1418201

**Published:** 2024-09-27

**Authors:** Jiyang Chen, Jianwen Guo, Hewei Zhang, Zhixiang Liang, Shuai Wang

**Affiliations:** Dongguan University of Technology, Dongguan, China

**Keywords:** plant disease, deep learning, Siamese networks, weakly supervised localization, class activation mapping

## Abstract

**Problems:**

Plant diseases significantly impact crop growth and yield. The variability and unpredictability of symptoms postinfection increase the complexity of image-based disease detection methods, leading to a higher false alarm rate.

**Aim:**

To address this challenge, we have developed an efficient, weakly supervised agricultural disease localization model using Siamese neural networks.

**Methods:**

This model innovatively employs a Siamese network structure with a weight-sharing mechanism to effectively capture the visual differences in plants affected by diseases. Combined with our proprietary Agricultural Disease Precise Localization Class Activation Mapping algorithm (ADPL-CAM), the model can accurately identify areas affected by diseases, achieving effective localization of plant diseases.

**Results and conclusion:**

The results showed that ADPL-CAM performed the best on all network architectures. On ResNet50, ADPL-CAM’s top-1 accuracy was 3.96% higher than GradCAM and 2.77% higher than SmoothCAM; the average Intersection over Union (IoU) is 27.09% higher than GradCAM and 19.63% higher than SmoothCAM. Under the SPDNet architecture, ADPL-CAM achieves a top-1 accuracy of 54.29% and an average IoU of 67.5%, outperforming other CAM methods in all metrics. It can accurately and promptly identify and locate diseased leaves in crops.

## 1 Introduction

Disease detection in agriculture plays a crucial role in ensuring crop health and maximizing yields. Traditionally, manual inspection and experience-based judgment have been used to identify diseases, but these methods often lack efficiency and accuracy, particularly for minor or inconspicuous ailments. With the advancement of machine vision and deep learning models, particularly Convolutional Neural Networks (CNNs) (LeCun et al., 2015), significant progress has been made in computer vision techniques for agricultural disease detection (Ferentinos, 2018). Utilizing these cutting-edge technologies for disease image classification has greatly improved the accuracy and robustness of detection.

However, current deep learning vision detection models still face challenges when dealing with the diversity and randomness of plant diseases. For example, diversity can lead to poor adaptability of traditional algorithms at different scales, resulting in missed or false detections. Diseases might be difficult to detect due to variations in the size, shape, or color of plant leaves or due to environmental factors such as lighting and occlusion. Traditional CNN architectures often perform poorly in addressing these issues (Fuentes et al., 2017) as they are designed with fixed scales and field-of-view sizes, which do not adapt well to varying sizes of disease features, especially in large-scale agricultural fields. Moreover, conventional disease detection methods require extensive annotation of datasets, which increases training costs and limits application scenarios. In contrast, weakly supervised learning can effectively detect using existing image category labels, significantly reducing the reliance on detailed annotations. Current weak supervision localization techniques primarily rely on multiple instance learning (Carbonneau et al., 2018) and Class Activation Mapping methods (Zhou et al., 2016), which train networks using image-level labels but often focus only on local features, making it difficult to cover the entire target and handle multiple instances of the same category.

To address these challenges, we propose an innovative detection model based on Siamese neural networks and weak supervision localization techniques, transforming the disease detection problem into a task of visual difference identification. By integrating multiscale features and implementing a refined weighting strategy, we have enhanced the accuracy and efficiency of disease identification. We use the ADPL-Class Activation Map (CAM) technique to generate heatmaps for precise disease localization and employ Non-Maximum Suppression (NMS) technology to handle multiple case issues, effectively improving the model’s performance in complex environments.

The latter part of this article will detail the relevant research work, foundational knowledge of Siamese networks and Class Activation Mapping techniques, describe our model architecture and experimental design, and demonstrate the effectiveness of our model through experimental results. We will discuss these results, emphasizing their significance in the field of intelligent agricultural disease detection, and outline future research directions.

## 2 Related work

### 2.1 Advances in plant disease detection research using deep learning

Early-stage plant diseases refer to diseases or diseases that occur in the early stages of plant growth, usually in the early stages after infection. Their symptoms may not be easily observed or recognized but may have potential impacts on the health and growth status of plants. The automatic recognition of early-stage plant disease images has traditionally relied on conventional machine learning techniques such as K-Nearest Neighbors (KNN) (Kumar et al., 2020), Support Vector Machines (SVM) (Rumpf et al., 2010), and Deep Forest methods (Zhou and Feng, 2017). However, with the advent of deep learning models, intelligent diagnostic methods based on these technologies have become the mainstream approach for image recognition (Sankaran et al., 2010) and have been increasingly applied to crops like corn, wheat, citrus, and potatoes (Ferentinos, 2018). For instance, (Mohanty et al., 2016) have demonstrated the accuracy and robustness of deep learning in classifying a vast array of plant disease images using CNNs. Similarly, the deep learning models developed by (Sladojevic et al., 2016) and the PlantXViT model introduced by Poornima et al (Poornima and Pushpalatha, 2021), which combines CNNs with Vision Transformers, have achieved notable success in plant disease recognition.

To address the shortage of datasets, researchers have explored small sample learning: (Li et al., 2023) investigated the potential of Diffusion Models (DDPM), Swin-Transformer models, and transfer learning for diagnosing citrus diseases with limited datasets. (Lee et al., 2018) designed two new data generation methods based on plant canopy simulation and Generative Adversarial Networks (GANs), which successfully handled the challenging task of segmenting apple scab disease in apple tree canopy images, showing promising results on small datasets. In terms of transfer learning, (Atila et al., 2020) proposed an efficient network of deep learning models for classifying plant leaf diseases, trained using the transfer learning approach on the EfficientNet architecture and other deep learning models. (Zj et al., 2019) enhanced the VGG16 model with multitask learning concepts and then applied transfer learning with pretrained models from ImageNet, effectively recognizing diseases in rice and wheat leaves, and providing a reliable method for identifying multiple plant leaf diseases. (Chen et al., 2018) explored deep convolutional neural network transfer learning to identify plant leaf diseases, considering using pretrained models from large-scale datasets and then transferring them to specific tasks.

Deep learning models still face challenges in handling the multiscale and randomness aspects of diseases. Diseases may appear on plants in various sizes, shapes, and colors, making it difficult for traditional algorithms to adapt to different scales and potentially leading to missed or false detections. Additionally, the same disease might appear differently on various plants and be influenced by environmental factors such as lighting and occlusion, increasing the likelihood of false positives. This presents significant challenges for disease detection, especially in large-scale agricultural environments. To overcome these issues, new solutions are being explored: (Singh et al., 2018) used Long Short-Term Memory (LSTM) networks (Hochreiter and Schmidhuber, 1997) to detect moisture stress in chickpea bud images, showcasing the potential of LSTM networks in multiscale diagnosis. (Mahlein, 2016) discussed methods for plant leaf disease detection using imaging sensors, highlighting the randomness in disease manifestation and proposing solutions. (Sumaya and Uddin, 2021) emphasized the importance of using deep learning for multiscale diagnosis and made progress in diagnosing various plant leaf diseases.

We believe that traditional CNN architectures, designed with predetermined image scales (He et al., 2016) and fixed receptive fields, struggle to adapt to disease spots of varying sizes (Liu et al., 2016). Additionally, these networks are not well-suited for spatial transformations such as rotation and scaling (Jaderberg et al., 2015), which can vary significantly across different plants, resulting in poor performance in such tasks. Moreover, these networks may lose crucial detailed information necessary for identification while extracting high-level semantic information (Zeiler and Fergus, 2014).

### 2.2 Siamese network

**Figure 1:**
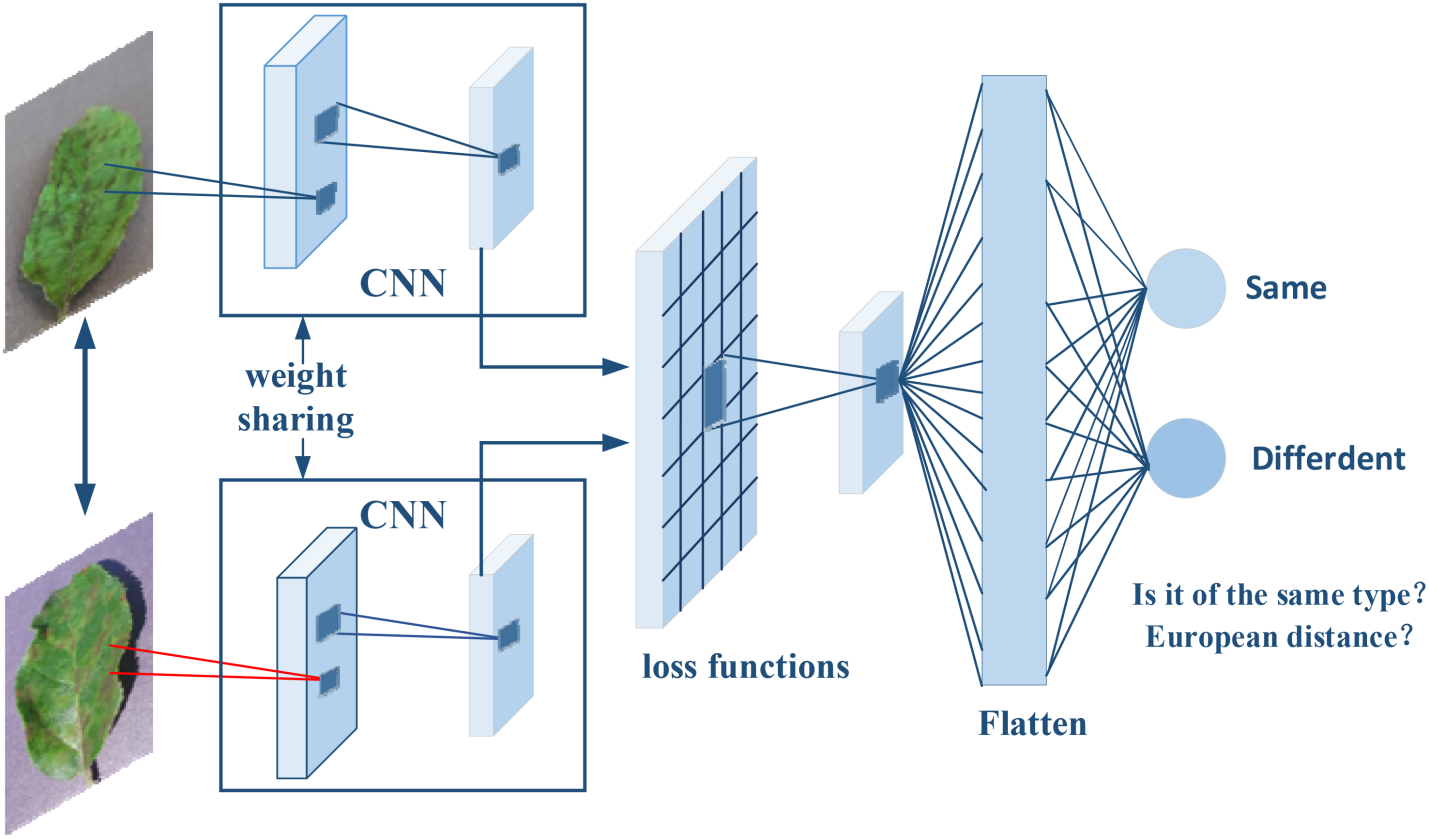
The architecture of the Siamese network was used in this study. The network independently analyzes each input image and deconstructs it into a detailed array of features such as lines, edges, textures, and patterns. These features are then transcribed into vectors, representing the images’ visual characteristics. The network is trained with pairs of known similar images to enhance its proficiency in detecting fine distinctions and shared characteristics.

The Siamese network, as illustrated in **Figure 1**, is a specialized neural network architecture designed for image comparison and verification tasks. This architecture is characterized by its two parallel branches, mirroring each other and sharing identical parameters, much like the interconnected nature of Siamese twins—hence the name. The primary benefit of this shared-parameter design is that it ensures both branches carry out the same transformations. Consequently, each input image is transformed into a feature vector, enabling a direct and equitable comparison.

To enhance the network’s ability to accurately measure image similarity, loss functions such as ContrastiveLoss (Hadsell et al., 2006) and TripletLoss (Schroff et al., 2015) are employed during the network’s training phase. These functions are crucial for the fine-tuning of network parameters, directly impacting the precision of similarity measurements.

Upon inputting two distinct photographs, the network analyzes each one independently. Each branch meticulously deconstructs its image into a detailed array of features—lines, edges, textures, and patterns—that define the image’s unique identity. These features are then transcribed into vectors, comprehensive numerical sequences that represent the images’ visual characteristics. Thanks to the Siamese configuration, this feature extraction process is consistently executed across both branches, laying the foundation for a balanced comparison.

Siamese networks have proven their effectiveness in a spectrum of applications. For instance, the DeepFace system (Taigman et al., 2014) harnesses a Siamese network for facial recognition, demonstrating its prowess in complex identification tasks. Similarly, the SiamFC tracker (Bertinetto et al., 2016) showcases the power of Siamese networks in real-time object tracking in video streams. Beyond these, the architecture has shown exceptional performance in recognizing Chinese handwritten characters (Zhang et al., 2017) and evaluating semantic similarity in natural language processing (Mueller and Thyagarajan, 2016).

In our research, we leverage the Siamese network’s dual-branch feature extraction capability by inputting image pairs that exhibit similarity. This approach allows us to produce highly accurate feature maps that are essential for precisely pinpointing object locations within images. By training the network with pairs of known similar images, we enhance its proficiency in detecting fine distinctions and shared characteristics between images, which is critical for tasks that demand exact localization.

### 2.3 Class activation map

**Figure 2:**
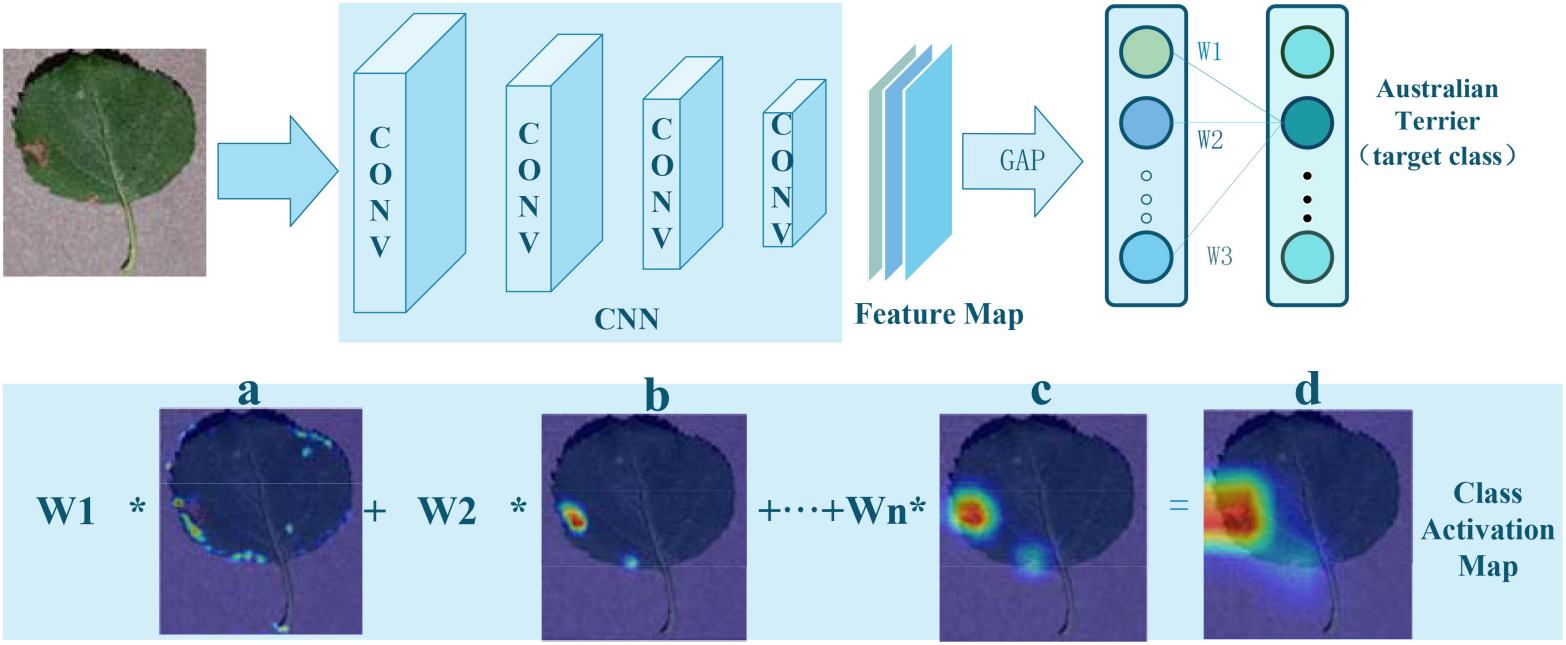
Implementation of Class Activation Mapping: **(A–C)**, CAM color heatmaps; **(D)**, original image overlaid with the CAM heatmap.

The CAM is a technique used in imaging to interpret and visualize the decision-making process of CNNs. It is based on a critical insight: classification networks not only extract categorical information from images but also implicitly encode spatial location information of targets. CAM generates heatmaps for specific categories by combining the outputs of a Global Average Pooling (GAP) layer with the feature maps from the last convolutional layer, visually indicating the target locations. The implementation process is illustrated in **Figure 2**.

Initially, an input image is processed through a CNN, producing a set of feature maps. Following the last convolutional layer of the CNN, a GAP layer is employed to calculate the average activation of each feature map, as shown in Equation 1:


(1) $$\begin{array}{c} F_{k}=\frac{1}{H\times W}\sum_{i=1}^{H}\sum_{j=1}^{W}f_{ij}^{k} \end{array}$$

Here, $f_{ij}^{k}$ denotes the activation value at position (*i*, *j*) on the *k*th feature map, with *H* and *W* representing the height and width of the feature map, respectively.

Subsequently, the output from the GAP layer is connected to a fully connected layer, whose weight matrix is used to compute the scores for each category:


(2) $$\begin{array}{c} S_{c}=\sum_{k}W_{k,c}\cdot F_{k} \end{array}$$

In this formula, *W* is the weight matrix of the fully connected layer, $W_{k,c}$ represents the weight between the *k*th feature map and the *c*th category, and $\;F_{k}$ is the average activation of the *k*th feature map.

Finally, by multiplying each feature map’s activation values by their corresponding category weights and summing them up, a class activation map is generated:


(3) $$\begin{array}{c} M_{c}\left(i,j\right)=\sum_{k}W_{k,c}\cdot f_{i,j}^{k} \end{array}$$

This map is the same size as the original image and uses grayscale values to indicate the significance of different areas for the network’s prediction. Higher scores indicate greater contributions to the final classification outcome. Converting this grayscale map to a color map can more clearly show which parts of the image are most focused on by the network and which areas are most predictive for a particular category.

The CAM method has been successfully applied in many research tasks, such as using CAM to locate pneumonia in chest X-ray images (Wang et al., 2017). Researchers have developed several variants of CAM, such as Grad-CAM (Selvaraju et al., 2017), which uses category-specific gradient information to weight feature maps, extending CAM’s applicability to more CNN architectures. Score-CAM (Wang et al., 2020) and Layer-CAM (Jiang et al., 2020) enhance the usability and interpretative power of CAM methods through model scoring and specific layer visualization, respectively.

## 3 Plant disease localization model based on Siamese neural networks

The traditional backbone networks often struggle to adequately recognize the subtle variances present in crop disease symptoms. To address this issue, we present SPDNet, a Siamese neural network-based method for weakly supervised localization of plant diseases. SPDNet is ingeniously crafted to tackle the challenges associated with the nuanced differences in infection symptoms and the presence of multiscale features.

The SPDNet model begins by inputting pairs of images that exhibit similar plant disease symptoms, with each pair comprising a query image and a reference image. A Siamese neural network, initialized with shared parameters, processes both the query and reference images. The query image is fed into the first subnet to extract feature maps, while the reference image is processed through the second subnet for feature extraction. Subsequently, a pyramid structure is employed to fuse the multiscale feature maps obtained from both the query and reference images, ensuring a comprehensive representation of disease symptoms across different scales. These fused multiscale feature maps are then input into the ADPL-CAM-based weakly supervised localization module. This module autonomously generates pseudo-detection bounding boxes to identify potential disease symptom regions. Following this, the ADPL-CAM module’s localization results are used to predict bounding boxes around the disease symptoms in the query image, with the disease locations highlighted in red on the heatmap. The SPDNet model is trained using weakly supervised learning methods, leveraging pseudo-labels generated by the ADPL-CAM module instead of precise annotations. During the iterative training process, the model is continuously refined to improve accuracy in disease localization.

**Figure 3:**
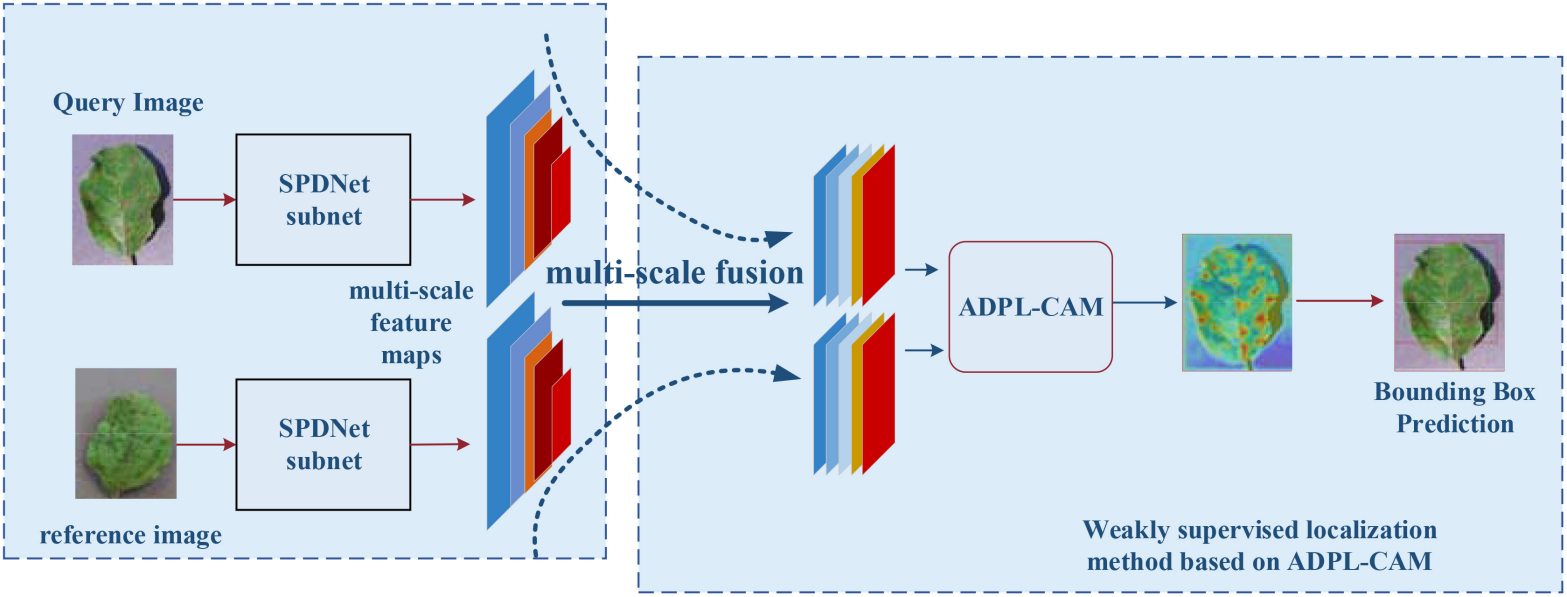
Flowchart of a weakly supervised plant disease localization model based on Siamese neural networks, where the blue represents the Siamese neural feature extraction network, and the red denotes the weakly supervised localization module based on ADPL-CAM. In the heatmap, red indicates the location of the disease.

The architecture of SPDNet, as illustrated in **Figure 3**, leverages shared parameters within its Siamese framework to enhance the model’s sensitivity to minor discrepancies between input images. The network processes pairs of disease images that share similar characteristics, using one image as the target and the other as a referential guide for localization. This dual-image input strategy enables SPDNet to develop more refined and distinctive feature representations, crucial for distinguishing between subtle disease symptoms.

Within the Siamese network, a pyramid structure is employed to amalgamate multiscale information extracted at various layers, ensuring a thorough representation of disease symptomatology across different scales.

The ADPL-CAM-based weakly supervised localization module is a core component of SPDNet, tailored for effective internal feature mapping during the detection and localization of plant diseases. It autonomously generates pseudo-detection bounding boxes, thereby diminishing the dependency on precisely annotated data. This module’s capability to produce pseudo-labels is pivotal for the generation of bounding boxes and the execution of weakly supervised localization tasks.

The employment of weakly supervised learning methodologies is a strategic choice for training SPDNet models. Given the laborious and sometimes unfeasible nature of acquiring fully annotated datasets in the agricultural domain, the weakly supervised approach is exceptionally pertinent. It facilitates the training of SPDNet with a reduced need for meticulously labeled data. The generation of pseudo-labels by SPDNet’s localization module acts as a surrogate for detailed annotations, making the training process more scalable and economically viable while preserving effectiveness. Through the study of SPDNet, we have reduced the dependence on precisely annotated data, which enables it to work effectively even in situations where annotated data are scarce, breaking free from the limitations of supervised learning methods like PiTLiD (Liu and Zhang, 2022) on small sample datasets.

### 3.1 SPDNet Siamese network development

**Figure 4:**
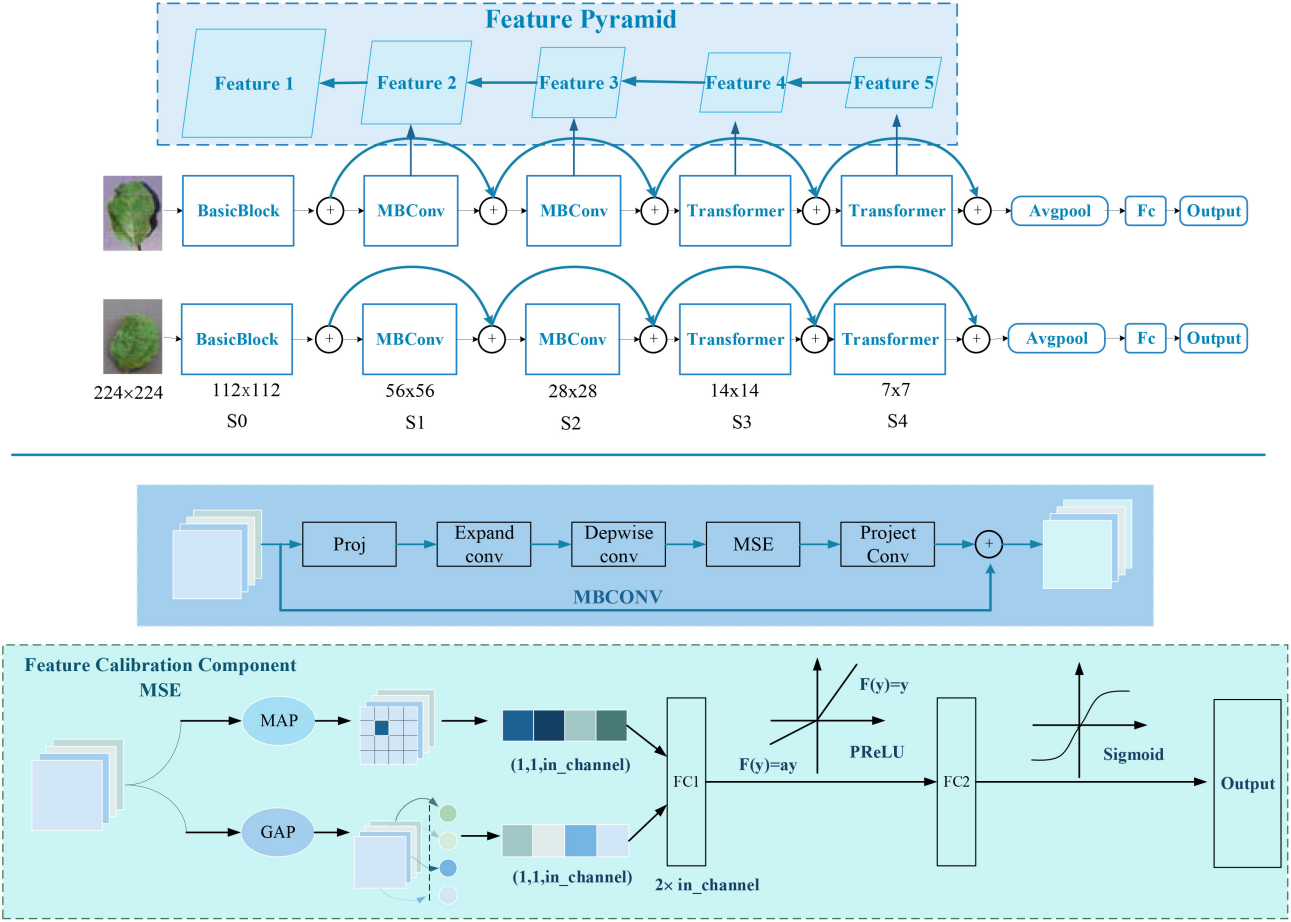
Architecture diagram of the SPDNet Siamese network component.

The development of the SPDNet Siamese network aims to overcome a series of challenges faced by traditional CNNs when processing crop disease images, particularly issues related to handling multiscale image features, adapting to spatial transformations like rotation and scaling, and preserving detailed information. The SPDNet employs a dual-branch structure to extract complementary features, which effectively deals with spatial transformations in disease areas and enhances the robustness of localization results. The architecture of the SPDNet Siamese network is shown in **Figure 4**, featuring this dual-branch structure.

The feature extraction part of the network utilizes a Feature Pyramid Structure (Lin et al., 2017), a strategy for extracting and integrating information across multiple scales. This allows for a comprehensive capture of disease symptom features of varying sizes. By merging features across scales, the network adaptively responds to changes in the size of disease areas, enhancing the robustness of the localization outcomes. In the higher layers, SPDNet incorporates both GAP and Global Max Pooling (GMP) (Zhou et al., 2016) to fuse features, which highlights the most significant features while also considering the average characteristics of the images, thus balancing global and local information. Moreover, SPDNet introduces a Multi-Scale Excitation (MSE) module to boost its representational power by adaptively adjusting the weights of different feature channels, focusing on the most pertinent features. The network also includes Parametric Rectified Linear Unit (PReLU) (He et al., 2015) as a nonlinear activation function and a dropout mechanism (Srivastava et al., 2014) for regularization, further enhancing the network’s learning capabilities and feature robustness.

#### 3.1.1 Detailed component descriptions

##### 3.1.1.1 Basic block

This consists of a 3 × 3 convolution, batch normalization, and ReLU activation:


(4) $$\begin{array}{c} y=\text{ReLU}\left(\text{BN}\left(\text{Conv}\left(\text{x}\right)\right)\right) \end{array}$$

where Conv represents 3 × 3 convolution, BN denotes batch normalization, and ReLU is the activation function.

##### 3.1.1.2 Feature Calibration Component MSE

The Feature Calibration Component MSE (Hu et al., 2018) facilitates the modeling of the importance across different semantic feature channels. By utilizing GAP and GMP to extract the average vector vavg and maximum vectorvmax, respectively, and then concatenating them along the channel dimension, the resulting vector is input into a fully connected network to learn channel correlations.

The computation of the channel attention vector is formulated as follows:


(5) $$\begin{array}{c} Z=\left[GAP\left(x\right);GMP\left(x\right)\right] \end{array}$$

(6) $$\begin{array}{c} A=\sigma \left(W_{2}\left(\delta \left(W_{1}Z\right)\right)\right) \end{array}$$

where σ denotes the Sigmoid function, δ represents the PReLU activation function, [;] indicates the concatenation operation, and *W*1 and *W*2 are learnable weights.

##### 3.1.1.3 More detailed structure and parameter selection

GAP and GMP are employed to compress each channel of the input feature map into a single scalar value, representing the global average and global maximum of that channel, respectively. The pooled features (concatenated results of GAP and GMP, with a dimension twice the number of input channels) are mapped to a hidden layer. The hidden layer’s channel count is set to 25% of the input channel count (controlled by the expansion parameter). The weights of the first fully connected layer (FC1) are initialized using the He initialization method. Batch normalization is applied to stabilize the training process. Dropout is used to prevent overfitting, with the dropout rate set to 0.5. The PReLU activation function is applied after the first fully connected layer. The Sigmoid activation function is applied after the second fully connected layer, compressing the output values to the range [0, 1]. The weights of the second fully connected layer (FC2) are initialized using the Xavier initialization method.

##### 3.1.1.4 MBConv module

A mobile-optimized bottleneck residual block structure that introduces the MSE mechanism between input and output (Sandler et al., 2018):


(7) $$\begin{array}{c} x\leftarrow \text{ReLU}\left(\text{BN}\left(\text{DWConv}\left(\text{ReLU}\left(\text{BN}\left(\text{Expand}\left(\text{x}\right)\right)\right)\right)\right)\right) \end{array}$$

(8) $$\begin{array}{c} attention=\sigma \left(MSE\left(x\right)\right) \end{array}$$

(9) $$\begin{array}{c} \;x\leftarrow x+Proj\left(x⊙\text{attention}\right) \end{array}$$

where Expand represents channel expansion via 1x1 convolution, DWConv stands for depthwise separable convolution, and Proj is a 1 × 1 convolution projection.

##### 3.1.1.5 Transformer module

Based on a conventional Attention and FFN transformer encoder structure, the main process involves MST, LayerNorm, Attention computation, and residual connections (Vaswani et al., 2017):


(10) $$\begin{array}{c} x_{1}=MST\left(x\right) \end{array}$$

(11) $$\begin{array}{c} z_{1}=Attention\left(LN\left(x_{1}\right)\right)+x_{2} \end{array}$$

(12) $$\begin{array}{c} z_{2}=FFN\left(LN\left(z_{1}\right)\right)+z_{1} \end{array}$$

where ×2 is a downsampling or equivalent Identity, LN denotes LayerNorm normalization, and MST represents multiscale integration of different sampling information.

##### 3.1.1.6 Feature Pyramid Structure

After extracting features at each level, a 1 × 1 convolution processes internally before upsampling is combined with the previous layer’s feature map, and a 3 × 3 convolution smoothly integrates to ensure consistent output scale and channel number (Lin et al., 2017):


(13) $$\begin{array}{c} C_{i}=Conv_{1\text{x}1} \end{array}$$

(14) $$\begin{array}{c} P_{i}=Upsample\left(P_{i+1}\right)+C_{i} \end{array}$$

(15) $$\begin{array}{c} FPN_{i}=Conv_{3\times 3}\left(P_{i}\right) \end{array}$$

By employing a complex design with multiple modules operating at different sampling rates, the SPDNet Siamese network not only captures disease features across various scales but also effectively minimizes localization errors due to changes in disease appearance through its dual-branch structure’s complementary characteristics, demonstrating exceptional performance.

### 3.2 Weakly supervised localization based on ADPL-CAM

**Figure 5:**
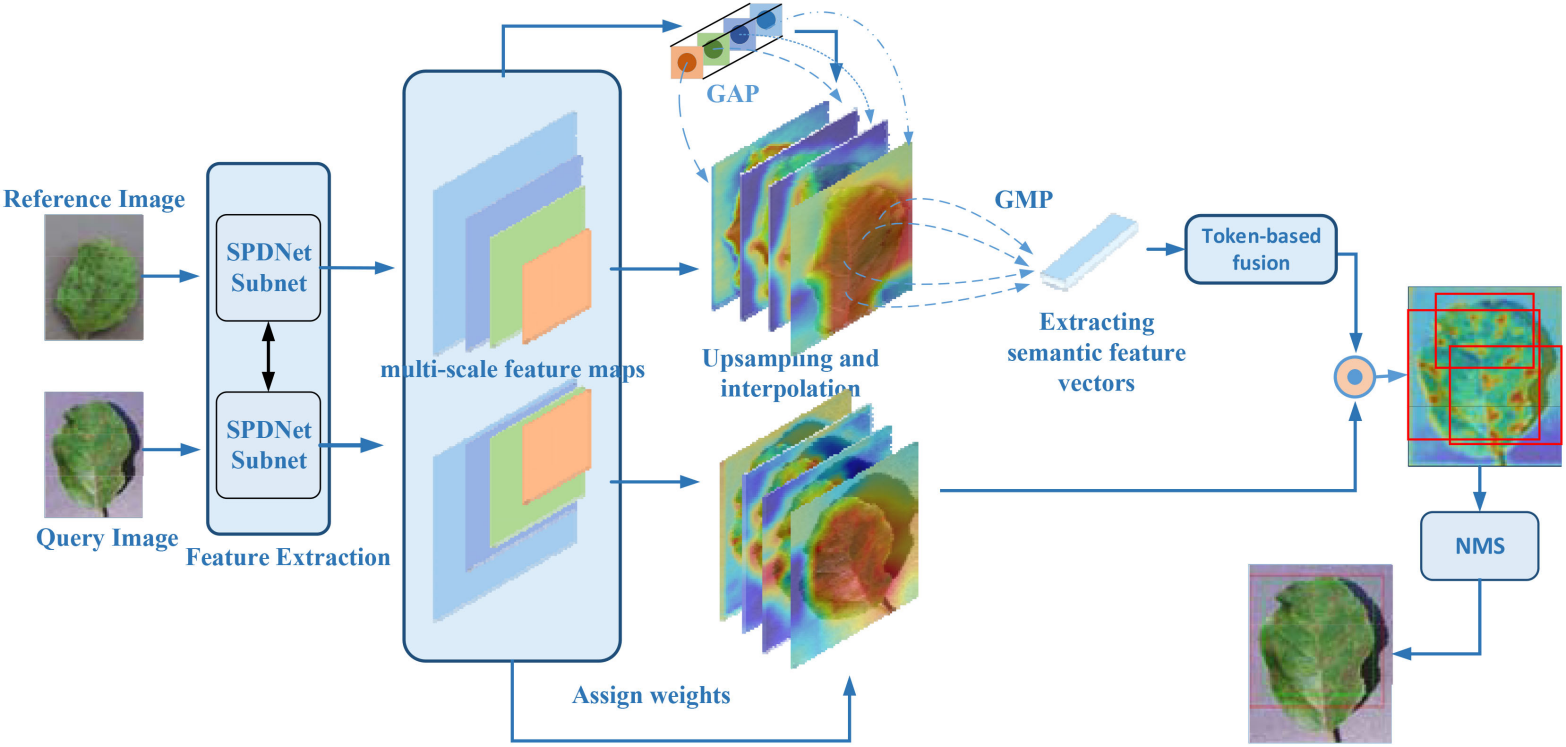
ADPL-CAM workflow diagram. Feature extraction: Feature maps are extracted in parallel from the reference and query images via SPNet subnetworks. Hierarchical weight activation: Emphasizes or attenuates the importance of certain features through the network layers. Feature tokenization: The feature maps from the reference image undergo tokenization, transforming these features into a set of compact tokens. Token-based fusion: Tokens from the reference image are fused with the feature maps of the query image, enhancing the feature representation of the query image. Class Activation Mapping: Postfusion, a sequence of processing steps generates the class activation map, highlighting areas of interest in the query image. Non-Maximum Suppression (NMS): To conclude, NMS is applied to the class activation map to suppress overlapping detections, ensuring distinct localization of each detected object.

To enhance the accuracy and robustness of disease symptom localization in SPDNet, this study introduces an innovative Class Activation Mapping method named Agricultural Disease Precise Localization Class Activation Map (ADPL-CAM). The overall detailed workflow diagram is shown in **Figure 5**. This method was developed with an understanding of the limitations of traditional CAM technologies in handling agricultural disease images, especially their inadequacies in dealing with multiscale features and background noise. It utilizes multiscale feature maps generated by the two branches of the SPDNet Siamese network. Based on a pair of similar image inputs, categorized into a reference image and a query image (the actual target frame output image), where the reference image enhances the features of the query image. ADPL-CAM extracts two feature matrices and effectively merges feature maps from both branches using upsampling and interpolation methods.

Subsequently, these feature maps undergo pooling to activate hierarchical weight, using weights to absorb the importance of features from different network layers. Ultimately, ADPL-CAM undertakes token learning for the reference image’s features: employing global maximum pooling to extract semantic information (i.e., tokens) and then fusing these tokens with the feature maps of the query image. Through token-based fusion, the activation map of the query image prominently represents similar semantic features. This strategy not only intensifies the model’s focus on the disease target areas but also significantly reduces its sensitivity to background noise.

Moreover, ADPL-CAM incorporates a NMS strategy to optimize the generation of localization boxes. NMS identifies the local maxima within each potential target area and filters out areas with low scores or high overlap through thresholding, thus enabling more accurate delineation of disease areas and effectively reducing misses. This strategy is particularly aimed at localization challenges in scenarios where similar diseases are clustered, greatly enhancing the model’s precision and adaptability in complex agricultural settings.

#### 3.2.1 ADPL-CAM multiscale feature map-weighted fusion

The CAM is formulated as a weighted sum of feature maps:


(16) $$\begin{array}{c} CAM=\sum_{i=1}^{N}w_{i}\cdot F_{i} \end{array}$$

where *N* is the number of feature maps, $w_{i}$ are weights obtained via the global average pooling layer, and $\;F_{i}$ is the feature map at that scale.

#### 3.2.2 Token-based feature learning

Initially, we define the tokenization process for the reference image’s feature maps (feature tokenization) to extract representative feature vectors $T_{i}$ :


(17) $$\begin{array}{c} T_{i}=GlobalMaxPool\left(F_{i}\right) \end{array}$$

Here, the GlobalMaxPool operation performs global maximum pooling, traversing each channel of the feature map and retaining only the maximum value per channel, thus forming a compact feature vector. This vector $T_{i}$ acts as a token, capturing the most critical visual features. Subsequently, we fuse the target feature map G with the token ( $T_{i}$ ), resulting in an enhanced feature map:


(18) $$\begin{array}{c} G^{\prime }=G+\sum_{i=1}^{N}\alpha \cdot T_{i} \end{array}$$

where α represents the learned weights, indicating the contribution of different tokens to the target feature map.

#### 3.2.3 Adaptive threshold function for generating box thresholds


(19) $$\begin{array}{c} T\left(x,y\right)=\frac{1}{blocksize^{2}}\sum_{i,j\in neighborhood}I\left(i,j\right)-C \end{array}$$

Here (Bradley and Roth, 2007), T(x,y) is the threshold at the pixel location (*x*, *y*), *I* (*i*, *j*) is the value of the pixels in the neighborhood, *C* is a constant used to adjust the threshold, and blocksize squared represents the size of the neighborhood considered for local threshold computation.

#### 3.2.4 Non-maximum suppression

Define a set of detection boxes (*D* = *d*1,*d*2,…,*dn*), each with a corresponding confidence score (si), select the box (*d*max) with the highest score from (*D*). Calculate the Intersection over Union (IoU) with (*d*max) for the other boxes and remove those with high overlap. Repeat this process until only one box remains.

Thus, ADPL-CAM not only enhances the handling of multiscale features but also improves the accuracy of disease symptom localization, providing robust technical support for precise agricultural disease diagnosis.

## 4 Experiments and results

### 4.1 Experimental design

The model’s effectiveness is assessed using three main metrics: Top-K Positioning Accuracy, GT-Known Positioning Accuracy, and Average Intersection over Union (Average IoU).

Top-K Positioning Accuracy is defined as the condition where the correct category is among the top-K categories predicted by the model and the IoU between the model’s predicted bounding box and the actual bounding box exceeds a specified threshold (set at 0.5). If these conditions are met, the prediction is considered correct.

GT-Known Positioning Accuracy measures whether the model can accurately locate the object when the true category is known. The prediction is deemed accurate if the IoU between the predicted and actual bounding boxes exceeds a predetermined threshold.

Average IoU calculates the mean IoU value between all predicted and actual bounding boxes across all test images to gauge the model’s overall localization precision.

We selected Top-K Positioning Accuracy, GT-Known Positioning Accuracy, and Average IoU as our principal metrics for evaluation due to their recognized efficacy and standardization in assessing both classification and localization performances within the field of computer vision. Top-K Positioning Accuracy holds particular significance for applications in the real world, where the ability to generate multiple plausible predictions is often more beneficial than pinpoint accuracy in classification. This metric ensures that the correct category is listed among the top contenders, while the associated IoU threshold criterion guarantees precise object localization within the imagery—a critical factor for practical implementations such as precision agriculture or automated wildlife monitoring.

GT-Known Positioning Accuracy is deployed to gauge the model’s proficiency in object localization when the true category is pre-identified—a typical training and tuning scenario for models engaged in detection tasks. This metric singularly focuses on and evaluates the model’s spatial discernment capabilities. The Average IoU, on the other hand, extends to provide a cumulative measure of localization accuracy across all tested instances, offering insight into the model’s generalization capabilities across a diverse array of categories and conditions. By integrating these tripartite metrics, we ensure a holistic evaluation of the model’s competence in not just accurately classifying objects but also in their precise localization, both of which are indispensable for the practical deployment of such models in scenarios where accurate identification and exact object placement are of paramount importance.

In this work, we used two datasets: to further explore the adaptability of the model to changes in different lighting conditions, crop varieties, and disease stages, we constructed a Multi-Conditional Plant Disease Dataset (MCPDD) based on the PlantVillage dataset. This dataset generates image data for different lighting conditions, crop varieties, and disease stages through image processing and classification, specifically for plant disease detection research. MCPDD contains a total of 42 images of different types and degrees of diseases on grape, potato, and tomato leaves under different lighting conditions. This diversity meets the requirements of plant disease detection at different stages, ensuring full consideration of the subtle semantic features of early diseases.

In contrast, the CUB-200 dataset is a fine-grained image classification dataset focused on various animal species. The ADPL-CAM method leverages its capability to capture semantic features within the same class in images. The CUB-200 dataset is not only informative but also serves as a universal benchmark for fine-grained classification tasks. Therefore, evaluating the ADPL-CAM method on this dataset not only validates its overall effectiveness in capturing similar semantic features and generating accurate localization maps but also reaffirms its robustness in fine-grained classification tasks.

This study assesses the feature semantic extraction capabilities of ADPL-CAM using both the PlantVillage and CUB-200 datasets to comprehensively verify the method’s universality and effectiveness. The simulation experiments were conducted on a computer equipped with an RTX A5000 GPU and 24GB VRAM. The experimental environment included PyTorch 1.11.0, CUDA 11.6, cuDNN 8.4.0, and Python 3.9.12. Images were resized to 224 pixels × 224 pixels, and data augmentation techniques such as random rotation and Gaussian blur were applied. The training was performed using the AdamW optimizer with an initial learning rate of 0.01, a minimum learning rate of 0.0001, and a cosine annealing learning rate schedule. The training lasted for 100 epochs with a batch size of 16, and the experiments were conducted under consistent hyperparameter settings.

### 4.2 Quantitative experiments and discussion

**Figure 6:**
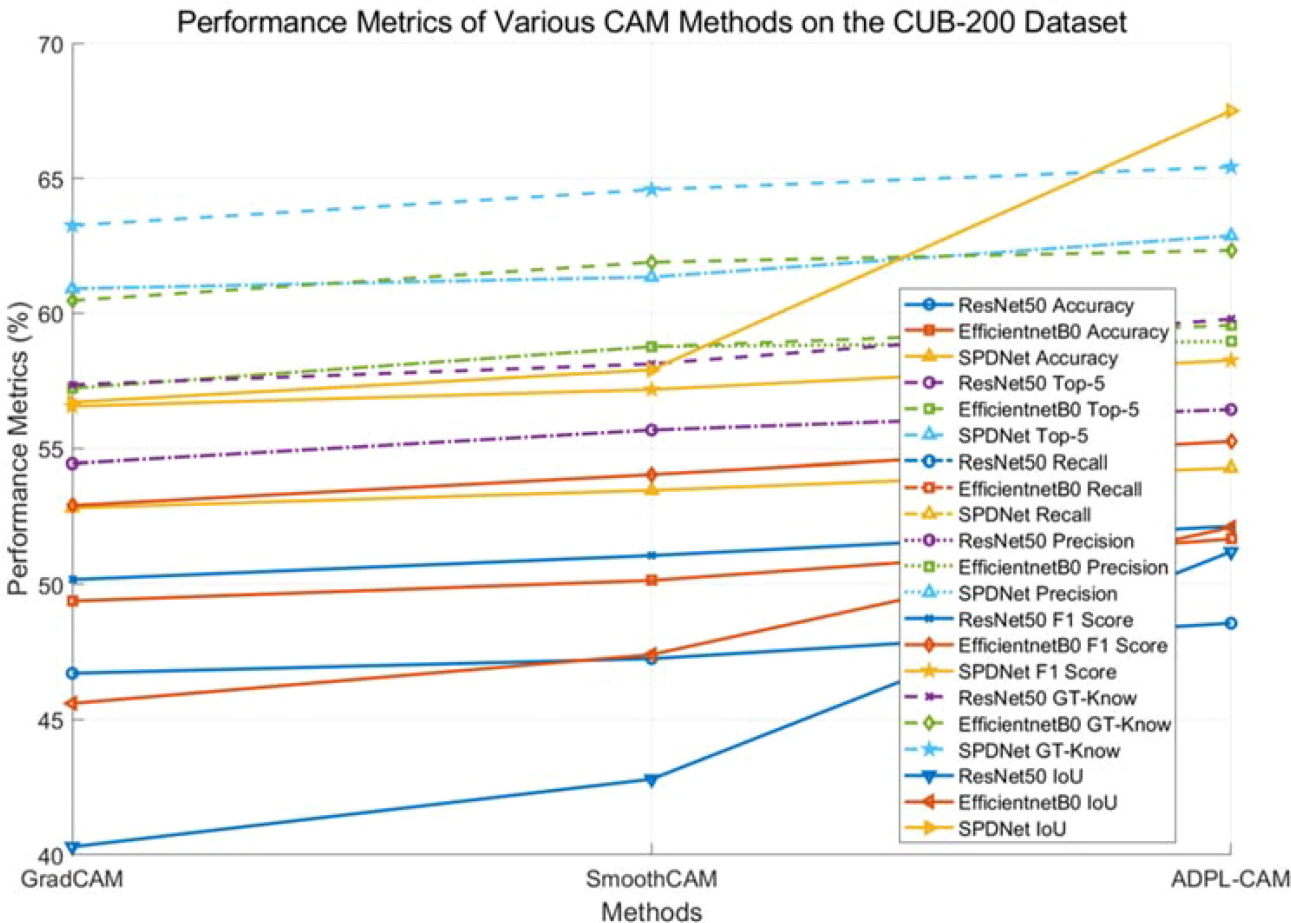
Displayed results of different visual models combined with different CAM methods on the CUB-200 dataset.

**Figure 7:**
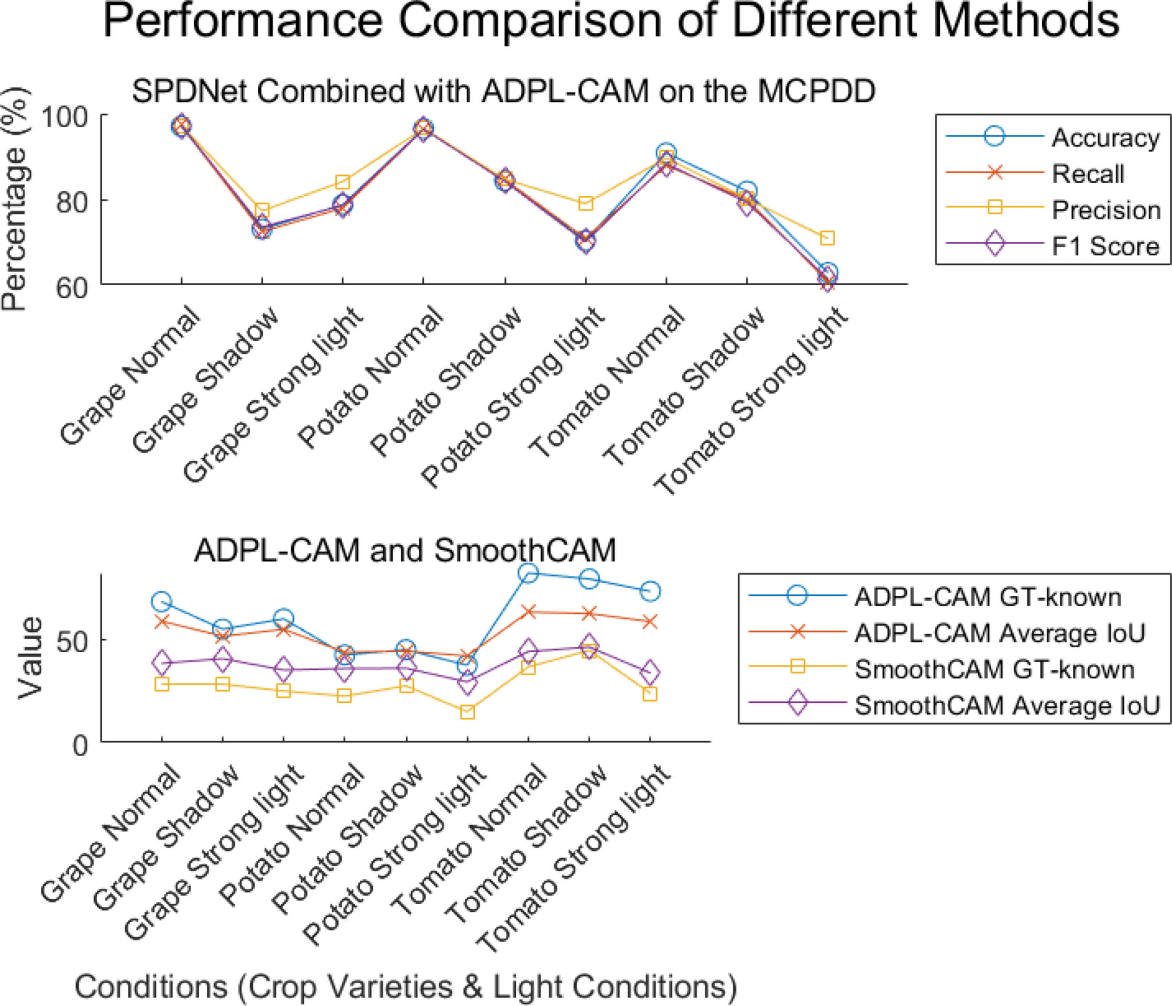
Visualization of experimental data. Displayed results of the combination of SPDNet and ADPL-CAM methods on MCPDD.

In our quantitative analysis, we used EfficientNet and ResNet50 as comparative classification networks and compared different CAM algorithms, including GradCAM, SmoothCAM, and our proposed ADPL-CAM. The results are shown in **Figures 6**, **7**, and detailed results are shown in **Tables 1**, **2**.

**Table 1:** Results of various CAM methods on the CUB-200 dataset (units: %).

Method	CNN	Top-1	Top-5	Recall	Precision	F1 score	GT-know	Average IoU
GradCAM	ResNet50	46.71	54.44	46.71	54.44	50.17	57.35	40.3
SmoothCAM	ResNet50	47.25	55.67	47.25	55.67	51.06	58.12	42.8
ADPL-CAM	ResNet50	**48.56**	**56.43**	**48.56**	**56.43**	**52.14**	**59.78**	**51.2**
GradCAM	EfficientnetB0	49.38	57.22	49.38	57.22	52.91	60.47	45.6
SmoothCAM	EfficientnetB0	50.14	58.76	50.14	58.76	54.05	61.89	47.4
ADPL-CAM	EfficientnetB0	**51.67**	**59.55**	**51.67**	**58.95**	**55.25**	**62.33**	**52.1**
GradCAM	SPDNet	52.82	60.91	52.82	60.91	56.55	63.25	56.7
SmoothCAM	SPDNet	53.47	61.34	53.47	61.34	57.17	64.58	57.9
ADPL-CAM	SPDNet	**54.29**	**62.87**	**54.29**	**62.87**	**58.25**	**65.42**	**67.5**

The bold values in the table indicate the optimal performance of each method on the CUB-200 dataset.

**Table 2:** Results of SPDNet combined with ADPL-CAM on the MCPDD.

Crop varieties	Light conditions	Accuracy	Recall	Precision	F1 score	GT-known (ADPL-CAM)	Average IoU (ADPL-CAM)	GT-known (SmoothCAM)	Average IoU (SmoothCAM)
Grape	Normal	97.09	97.34	97.30	97.30	68.33	58.90	28.33	38.39
	Shadow	73.06	72.47	77.31	73.53	55.00	51.56	28.33	40.68
	Strong light	78.77	77.96	84.16	78.57	60.00	54.90	25.00	35.16
Potato	Normal	96.50	96.35	96.55	96.40	42.50	43.79	22.50	35.88
	Shadow	84.33	84.62	84.69	84.24	45.00	44.32	27.50	36.03
	Strong light	70.33	70.88	79.00	70.04	37.50	42.04	15.00	29.42
Tomato	Normal	90.84	87.93	89.67	88.32	82.22	63.25	36.67	44.13
	Shadow	81.91	79.93	80.08	79.23	79.34	62.54	44.69	46.30
	Strong light	62.88	60.74	70.82	61.36	73.33	58.67	23.90	33.74

Based on the experimental results, we can draw the following conclusion.

1. Performance comparison: In the CUB-200 and PlantVillage datasets, ADPL-CAM outperformed Grad-CAM and SmoothCAM, especially within the SPDNet framework. Notably, under the SPDNet architecture, ADPL-CAM achieved the best results across all evaluation metrics (accuracy, recall, precision, F1-score, GT-known, and mean IoU). This demonstrates ADPL-CAM’s significant advantage in capturing salient regions of target objects and generating more accurate class activation maps.

2. Framework adaptability: The performance improvement of ADPL-CAM in fine-grained tasks when paired with ResNet50 and EfficientNetB0 is relatively modest. This can be attributed to these CNN architectures being primarily designed for general image classification tasks rather than specialized plant disease recognition. However, in the MCPDD dataset, ADPL-CAM’s performance is notably outstanding. This indicates that specifically designed network structures, such as SPDNet, can better capture task-specific features in specialized domains.

3. Disease recognition capability: The combination of SPDNet and ADPL-CAM shows significant advantages in plant disease recognition tasks, particularly in terms of various metrics. This suggests that SPDNet can effectively learn feature representations of plant diseases, contributing to more accurate localization maps. Traditional CAM methods (Grad-CAM and SmoothCAM) often perform poorly in complex or challenging disease scenarios, whereas ADPL-CAM maintains high effectiveness, which is crucial for improving model reliability in practical applications. ADPL-CAM excels in covering target areas more comprehensively. Through adaptive multiscale feature fusion and enhanced Class Activation Mapping mechanisms, ADPL-CAM can cover lesion areas more thoroughly, avoiding the omission of key features.

4. Performance deficiencies and potential factors: Despite ADPL-CAM’s improvement in overall localization accuracy, this experiment did not validate potential issues in complex scenarios, such as small or overlapping lesion areas, where the model might experience false negatives or misclassifications. The potential reason for this deficiency is that ADPL-CAM’s multiscale feature fusion mechanism requires further optimization to better leverage features at different levels. Although we have consciously enhanced fine-grained features in the dataset, the model appears not to have fully learned to recognize subtle disease characteristics. Label-based semantic enhancement may need improvement to distinguish disease samples with minor features. **Figure 7** also indicates that ADPL-CAM’s localization results are affected by factors such as illumination conditions and crop varieties. Among these, the most significant factor is crop variety, due to the vast semantic differences in characteristics of different plant diseases. Furthermore, ADPL-CAM’s generalization ability in small sample datasets might decline, necessitating further optimization of network structures and training strategies to enhance the model’s robustness in small sample scenarios.

### 4.3 Qualitative experiments and discussion

**Figure 8:**
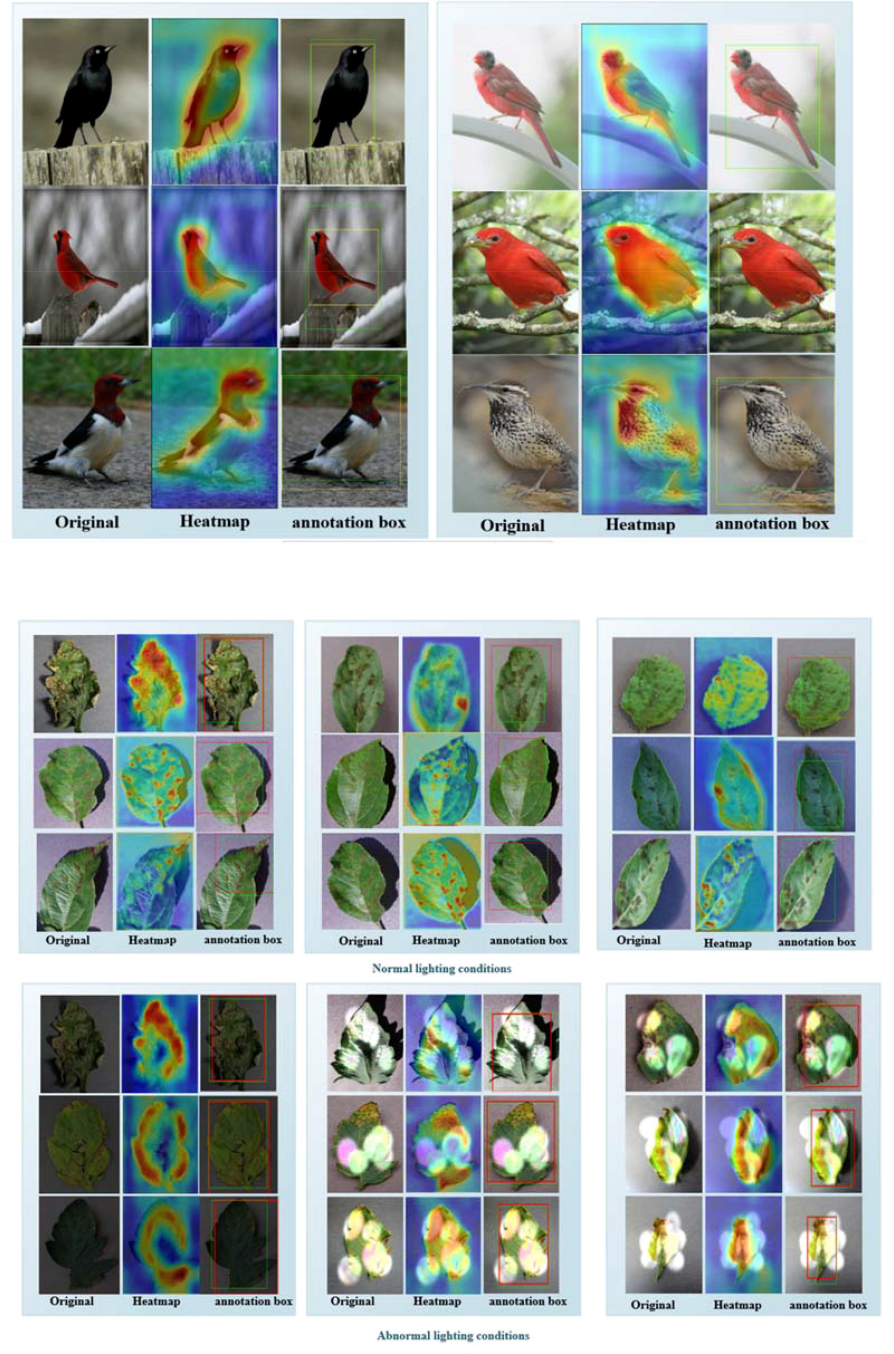
**(A)** Part of the experimental results on the CUB-200 dataset. The first column contains the original images, the second column shows the ADPL-CAM class activation maps, and the third column displays the localization maps. Yellow boxes represent the target boxes, while green boxes indicate the generated boxes. **(B)** Partial experimental results of the MCPDD dataset are described, with the first column being the original plant disease map, the second column being the ADPL-CAM class activation map, and the third column being the localization map. The green box represents the target box, and the red box represents the generated box.

We conducted our research using the SPDNet+ADPL-CAM strategy to visualize the effectiveness of our proposed method on two datasets and to compare the generated localization bounding boxes with the actual detection bounding boxes, as shown in **Figure 8**. Additionally, to provide a comprehensive display of this method’s performance, we have published all the localization data from our qualitative experiments on GitHub [Qualitative Experiment Visualization (github.com)].

By integrating the ADPL-CAM Class Activation Mapping method with the SPDNet architecture, a series of visualization results were obtained. These results demonstrate the potential advantages of this combination in feature recognition and target localization. From the visualized class activation maps, it is evident that this combination can accurately identify and locate target areas. This not only confirms the efficacy of SPDNet in capturing key features but also illustrates the capability of the ADPL-CAM method in accurately generating target localization frames (annotation boxes). This rapid target localization approach, based on image-level labels, offers significant advantages in reducing training costs and resource consumption. It also provides directions for further optimization of SPDNet and improvements to the ADPL-CAM algorithm.

However, the visualization results also highlighted some areas for improvement. When dealing with widely distributed and scattered disease features, ADPL-CAM tends to recognize only the most prominent parts, which could lead to failures in detecting multiple smaller features. Additionally, the detection outcomes are influenced by lighting conditions, which may affect the accuracy of the localizations.

## 5 Conclusion

This paper addresses the challenges of multiscale and random distribution of plant disease characteristics by proposing a weakly supervised localization model based on Siamese neural networks. This model is equipped with a proprietary ADPL-CAM algorithm, which accurately identifies and locates areas affected by plant diseases. In early-stage disease detection tasks, the model can timely and accurately identify and locate diseased crop leaves. Moreover, the model also demonstrates good performance in other feature recognition tasks. Delving deeply into the ADPL-CAM technology enhances our model’s capability to pinpoint plant diseases with remarkable precision. This empowers farmers with prompt and reliable diagnostic insights, mitigating the misuse of pesticides and avoiding the repercussions of misdiagnoses on crop yields. Enhancing the model’s resilience to fluctuations in light and extreme conditions is essential, guaranteeing consistent performance amidst the diverse and unpredictable agricultural landscapes. Integrated into an intelligent decision support framework, our model becomes a pivotal tool for farmers, aiding in the rapid identification of plant afflictions and offering strategic management advice, thereby diminishing labor demands and elevating agricultural productivity. Technicians benefit from the model’s swift disease detection, enabling them to tailor more effective control strategies, thus bolstering the efficacy of their interventions. For researchers, the model serves as a vigilant sentinel for disease surveillance and a robust data repository, laying down a solid scientific foundation for disease management and the cultivation of new crop varieties.

Future research will focus on the following areas:

1. Exploring ADPL-CAM mechanisms and mapping strategies: We plan to further investigate the mechanisms behind ADPL-CAM and its performance enhancement in various CNN architectures. This includes analyzing how it effectively integrates multiscale features and handles spatial transformations to optimize methods or develop more efficient CAM variants. Considering the limitations of ADPL-CAM in handling complex features, exploring new activation mapping techniques could be beneficial. For instance, introducing an attention-based Class Activation Mapping might help the model focus better on multiple key areas of the target.

2. Enhancing model robustness: Although ADPL-CAM maintains good performance in complex disease scenarios, enhancing the model’s adaptability to extreme variations (such as very small or concealed disease features) is also crucial. This might be achieved by integrating more fine-grained feature extraction mechanisms or using deeper learning strategies. The impact of lighting conditions on image recognition is a complex but critical issue. Model robustness to lighting variations could be improved through data augmentation (e.g., introducing a variety of lighting conditions during training) or by incorporating lighting-invariant features.

## Data Availability

The datasets presented in this study can be found in online repositories. The names of the repository/repositories and accession number(s) can be found below: https://AutoGo-Lab/SPDNet: Qualitative Experiment Visualization.
